# The Flow Engine Framework: A Cognitive Model of Optimal Human Experience

**DOI:** 10.5964/ejop.v14i1.1370

**Published:** 2018-03-12

**Authors:** Milija Šimleša, Jérôme Guegan, Edouard Blanchard, Franck Tarpin-Bernard, Stéphanie Buisine

**Affiliations:** a SBT Group, Paris, France; b LATI, University Paris Descartes, Paris, France; c LINEACT, CESI, Paris, France; Department of Psychology, Webster University Geneva, Geneva, Switzerland; University College Cork, Cork, Ireland

**Keywords:** flow, optimal experience, flow components, IPO model, cognitive processes, theoretical model

## Abstract

Flow is a well-known concept in the fields of positive and applied psychology. Examination of a large body of flow literature suggests there is a need for a conceptual model rooted in a cognitive approach to explain how this psychological phenomenon works. In this paper, we propose the Flow Engine Framework, a theoretical model explaining dynamic interactions between rearranged flow components and fundamental cognitive processes. Using an IPO framework (Inputs – Processes – Outputs) including a feedback process, we organize flow characteristics into three logically related categories: inputs (requirements for flow), mediating and moderating cognitive processes (attentional and motivational mechanisms) and outputs (subjective and objective outcomes), describing the process of the flow. Comparing flow with an engine, inputs are depicted as flow-fuel, core processes cylinder strokes and outputs as power created to provide motion.

Studying the creative process (Nakamura & Csikszentmihalyi, 2002), Csikszentmihalyi began to investigate a psychological phenomenon that he named *flow* (Csikszentmihalyi, 1993; Csikszentmihalyi, 2008; Csikszentmihalyi & LeFevre, 1989; Ghani & Deshpande, 1994). Flow corresponds to a state of optimal experience and maximal concentration, when people act at the peak of their capacity. It may lead to high levels of performance, creativity and pleasure. Encompassing specificities of various domains, a large variety of enjoyable human activities share the same flow characteristics (Csikszentmihalyi, 1994).

Csikszentmihalyi and other researchers found this experience by interviewing people that have left a significant trace in history with considerable achievements in literature, science, music, rock climbing, dancing, chess (Csikszentmihalyi, 2013); but also other domains such as sailing, line-work in industry (Csikszentmihalyi, 2008), and computer programming (Rogulja, Tomić, & Olčar, 2011). The account of the flow state is particularly robust, confirmed through numerous studies (Csikszentmihalyi, 2013; Csikszentmihalyi & Robinson, 1990; Perry, 1999). An eminent pianist performing in front of an audience could describe her psychological state as a fulfilling, absorbing experience of merging action and awareness while moving fingers across the keyboard, interpreting the piece and sharing beauty with her audience. If we ask a chess player how it feels when the tournament is going well, he will probably give a similar description like a pianist would give of a good concert.

Providing a promise for a full life worth living, flow both improves subjective well being and has a potential for socially useful consequences (Csikszentmihalyi, 1994). The more time that is spent in this state, the better the quality of life is: people experiencing flow report higher levels of concentration, creativity and positive emotions (Nakamura & Csikszentmihalyi, 2002). A wide range of empirical evidence indicates the adaptive importance of positive affects. Besides mere pleasure, positive affects bring numerous, interdependent benefits (Fredrickson & Losada, 2005). For example, positive feelings reshape people’s mindsets: research showed that induced positive affect stretches the scope of attention (Fredrickson & Branigan, 2005; Rowe, Hirsch, & Anderson, 2005), broadens behavioural range (Fredrickson & Branigan, 2005), boosts creativity (Isen, Daubman, & Nowicki, 1987), and increases intuition (Bolte, Goschke, & Kuhl, 2003).

Therefore, flow appears to be important for human well being, and its scientific understanding becomes a requisite for contributing to the improvement of human lives. Describing, explaining and predicting this phenomenon may help act upon and change behaviours for the best.

Three decades of empirical research on this topic have yielded results and insights about domain-related flow, notably music (e.g. Byrne, MacDonald, & Carlton, 2003; MacDonald, Byrne, & Carlton, 2006; Wrigley & Emmerson, 2013), sports (e.g. Catley & Duda, 1997; Kimiecik & Jackson, 2002; Stein, Kimiecik, Daniels, & Jackson, 1995), education (e.g. Bakker, 2005; Clarke & Haworth, 1994; Lee, 2005), video games (e.g. Bryce & Rutter, 2001; Cowley, Charles, Black, & Hickey, 2008; Thin, Hansen, & McEachen, 2011; Weibel, Wissmath, Habegger, Steiner, & Groner, 2008), work (e.g. Fullagar & Kelloway, 2009; Lavigne, Forest, & Crevier-Braud, 2012; Nielsen & Cleal, 2010), and other domains. These empirical studies assessed flow with standard measures such as experience sampling method or ESM (Csikszentmihalyi & Hunter, 2003; Csikszentmihalyi & Larson, 1987; Csikszentmihalyi, Larson, & Prescott, 1977; Hormuth, 1986; Larson & Csikszentmihalyi, 1983). This method consists in equipping the respondents with an electronic pager and a booklet of self-report forms. Participants wear the pager and whenever it beeps, they have to fill out a page of booklet indicating activity, location, companionship and the quality of experience at that moment on a variety of dimensions (task type, challenges and skills, quality of experience, affect, potency, concentration, creativity, motivation, satisfaction, relaxation, etc.). There are several other methods to measure flow such as The Flow Scale (Mayers, 1978), The Flow Questionnaire and Flow Scale (Delle Fave & Massimini, 1988), Activity Flow State Scale – AFSS (Payne, Jackson, Noh, & Stine-Morrow, 2011), Dispositional Flow Scale-2 (Jackson & Eklund, 2002), Flow Short Scale (Rheinberg, Vollmeyer, & Engeser, 2003), and some other paper-and-pencil scales used in sports (Jackson & Marsh, 1996) or psychotherapy (Parks, 1996).

## Characteristics of Flow

This state which allows individuals to achieve an ordered state of mind and that is highly enjoyable (Csikszentmihalyi, 2008) is characterised by the following features: (1) balance between perceived challenges and perceived skills, (2) clear proximal goals, (3) immediate feedback, (4) intrinsic motivation, (5) hyper focus, (6) temporary loss of reflective self- awareness, (7) distortion of time-perception, (8) feeling of control, and (9) merging of action and awareness (Nakamura & Csikszentmihalyi, 2002) to whom may be added a tenth characteristic (10) attentional-involvement (Abuhamdeh & Csikszentmihalyi, 2012a).

Hamari and Koivisto (2014) have suggested that flow should be regarded as divided between the conditions for reaching the flow and the psychological outputs that follow from reaching the optimal experience. Some flow dimensions are considered conceptually closer to one another. For example, theoritizations have considered challenge-skill balance, clear goals, control and feedback as conditions required to attain flow, while loss of self-consciousness, time distortion, concentration, and merging action-awareness have been regarded as outcomes (Csikszentmihalyi, 2008; Hamari & Koivisto, 2014; Nakamura & Csikszentmihalyi, 2002). Furthermore, evidence from psychometric data such as a stronger covariance between certain dimensions and weaker covariance between other dimensions, is coherent with the idea that there might be a possible conceptual diversity of flow dimensions (Boffi et al., in press; Fournier et al., 2007; Hamari & Koivisto, 2014).

Csikszentmihalyi (2014) seems to differentiate conditions (clear goals, skill-challenge balance, and immediate feedback), characteristics (concentration, merging action and awareness, loss of reflective self-consciousness, control, time distortion, and autotelic experience) and outcomes (persistence, commitment, achievement, less anxiety, etc.) of the flow experience. However, this differenciation between conditions, characteristics and outcomes was never directly put in perspective of a theoretical model. Similarly, Landhäusser and Keller’s (2012) model organizes the flow experience as a sequence of (1) preconditions (i.e., goals, feedback, demand-skill balance), (2) components of the experience (e.g., sense of control, reduced self-consciousness) and (3) consequences of flow (i.e. affective, cognitive, physiological, and quality of performance). Possible retroactions from the experience and consequences of flow onto the preconditions of further flow experience in an auto-alimentation phenomenon are not considered in this model. Moreover, cognitive functions are categorized as consequences of flow, which suggests that flow is viewed as a fully-fledged process emerging independently from them. Our approach mainly differs in two respects. Firstly, we believe that flow experience arises from the combination of favorable contextual factors (preconditions) and activation of specific cognitive functions (attentional and motivational processes) likely to mediate and/or moderate flow process. This may result in a more parsimonious and dynamic model drawing both on previous flow research, which has mainly taken place in domains of positive psychology and applied sciences (e.g., education, sports, information technologies and management), and on the framework of cognitive psychology. This attempt of linking flow to fundamental cognitive processes may also offer a conceptualization of flow inside, instead of beside, the domain of cognitive psychology.

Furthermore, the continuous evolution of challenge-skill balance refreshed by constant feedback and adaptation to changing proximal goals leads us to believe that flow is a dynamic psychological process, rather than a mere state. Happening in real-time, the task of the person experiencing flow provides a dynamic context of interaction between the doer, his/her environment and the activity. Already vividly described in literature (e.g., Csikszentmihalyi, 2008, 2013), at the present moment we lack a cognitive explanation of the flow process. Given these issues, we argue that a theoretical model describing the functional nature of flow is needed – giving a comprehensible explanation of this concept in a dynamic framework. Now that we are able to name, depict, notice, and recognize it, the next mandatory phase is explaining it. This indispensable step in studying psychological phenomena opens new possibilities for predicting flow and acting upon it. To our best knowledge, there haven’t been other attempts to produce a dynamic and cognitive conceptualization of flow.

## Our Theoretical Model

### The Flow Engine Framework

Just like an engine converts gasoline into motion, flow inputs are ignited by strokes of core processes, producing flow dynamics which consequently generates changes to the status quo: absorption, achievement and positive feelings. This theory seeks to provide a functional mechanism for the process of flow by using an I-P-O (Inputs-Processes-Outputs) framework adding retroaction loops. I-P-O models have demonstrated their utility in the context of empirical research (e.g., Campion, Medsker, & Higgs, 1993; Gladstein, 1984; Guzzo & Dickson, 1996) and they seem particularly appropriate to study causal systems in terms of mediating and moderating variables. In this respect, the analysis of mediators and moderators has long been recognized as fruitful in theoretical, strategic and statistical ways to offer a deeper comprehension of psychological phenomena (Baron & Kenny, 1986).

Inputs, the fuel of the flow engine, stand for conditions that exist prior to the task or so-called *performance episode.* Performance episodes can be defined as periods over which performance accrues and feedback is available, while processes stand for how inputs are transformed into outputs. Finally, outputs are all results and by-products of activity that are produced (Mathieu, Heffner, Goodwin, Salas, & Cannon-Bowers, 2000). This I-P-O model should not be understood literally as a strictly sequential, time dependent model. Rather, this should be taken as a logical structure allowing simultaneous change in parameters appearing in different structural sections, interdependency and feedback loops.

### I-P-O Flow Framework

**Figure 1:**
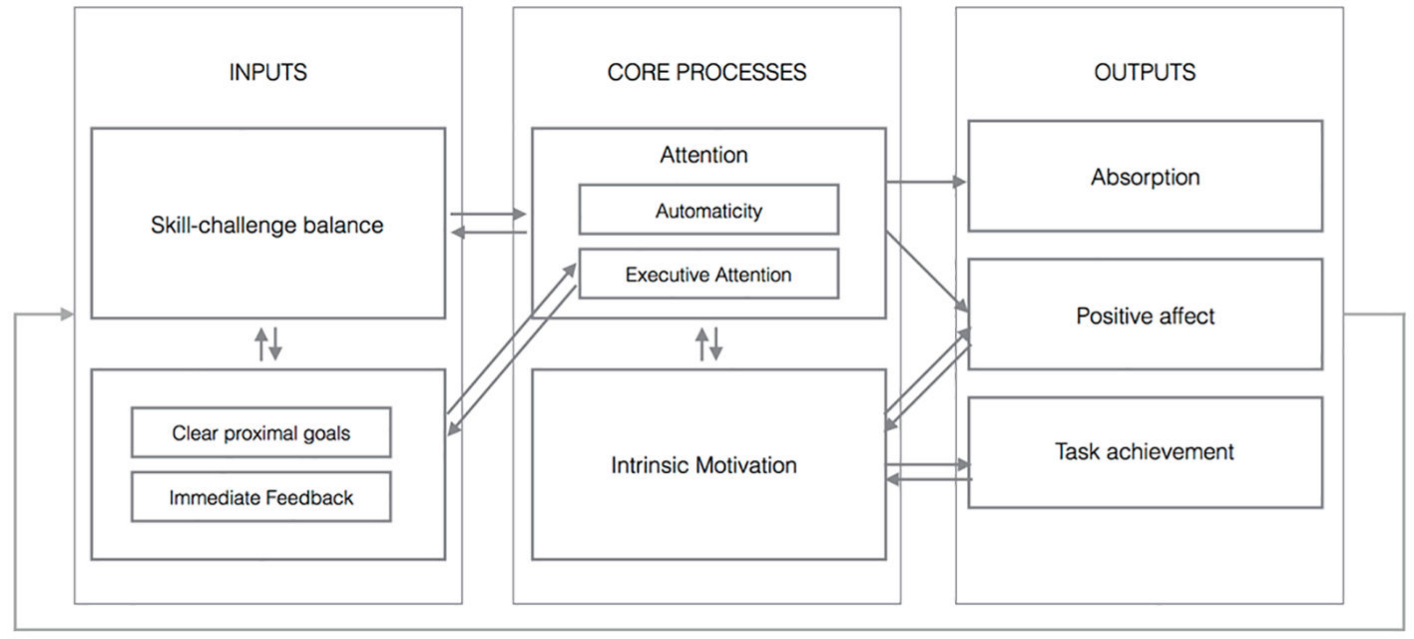
Flow Engine Framework. *Note.* The simple arrows represent causal relationships between elements. The double arrows represent the loops of interdependence.

The model consists of three structural sections: inputs, core processes and outputs. Among inputs, the I-P-O model incorporates (1) the skill-challenge balance, (2) clear proximal goals and immediate feedback. Core processes rely two key cognitive processes that are: (1) attention, and (2) motivation. Finally, outputs consist of three sets of flow outcomes: (1) subjective experience of absorption, (2) task achievements, the fruits of invested effort, and (3) positive affects (see Figure 1).

### Inputs

Inputs reflect the resources that individuals have at their disposal for entering the process of flow. Rather than chronological, those are time-independent dimensions that seem like logical pre-requirements for engaging in a flow-genic activity. Similarly to Landhäusser and Keller (2012), we posit that these inputs comprise: (1) challenge-skill balance, (2) clear proximal goals and immediate feedback, which are merged into a single precondition.

#### Balance Between Perceived Challenges and Perceived Skills

In order to get into the flow, a person’s perceived skills must match the perceived difficulty of the task – “a sense that one is engaging challenges at a level appropriate to one’s capacities” (Nakamura & Csikszentmihalyi, 2002). If the doer underestimates or overestimates his skills or challenges, reaching a state of flow is not possible. Playing a difficult piece that has not been practiced enough represents a big challenge. If the pianist does not have enough skills to overcome the challenges of the piece, the result will be a state of anxiety or even panic. On the contrary, if s/he is given pieces that are too simple, s/he risks falling into states of boredom and apathy. However, if the difficulty of the piece is corresponding to her skills (technique, work, practice, sensibility, etc.) the musician is likely to enter the zone of optimal experience. An initial balance between challenges and skills or a very slight misbalance between them (zone of control or zone of excitement) provides a starting point for an absorbing autotelic experience (meaning that it is done for the sake of doing rather than for the sake of something else). Without this pre-condition, there is no flow. For example, if perceived challenges are considerably superior to perceived skills, the person would be unable to invest his attention in the effective way, but will rather get lost in self-reflective rumination and sensations of anxiety.

#### Clear Proximal Goals and Immediate Feedback

The person experiencing flow needs clear proximal goals of where the action is leading him/her (Nakamura & Csikszentmihalyi, 2002), where he or she is heading and what the next step is.

Landhäusser and Keller (2012) argue that flow inputs can be simplified and reduced to perceived skills and challenge. Proximal goals refer here to small within-activity goals that arise out of the interaction and that are identifiable thanks to continuous feedback rather than the *structure of the task*. This means that the structure of the task is unfolding during the experience itself. Depending on the task, it can be more or less transparent and visible. For example, while playing a known piece, the musician will have a clearer view of the structure of the task (meaning the sequence of proximal goals). On the contrary, a skier on a new slope will have a less transparent image of the sequence of his proximal goals. For this reason, we reckon that the component of clear proximal goals should be maintained separated from skill-challenge balance even though they are obviously very much related. In the context of a musical interpretation, clear proximal goals can be translated in terms of expressing a certain emotion in a given sequence, or giving a certain colour to a staccato that is supposed to depict grasshoppers. Clear proximal goals allow certain cognitive and conative unburdening to the person so that his or her emergent long-term goals do not encumber her or his consciousness while doing the task. Ergo, these small proximal goals are indirectly related to motivational process as well.

In our view, clear proximal goals and immediate feedback are gathered in a single input because we consider them as closely interdependent: proximal goals may not be perceived without feedback on the activity, and immediate feedback may contribute to triggering flow only in conjunction with clear proximal goals. Like a signal that is looped back to update a process within itself, immediate feedback on an activity progression is necessary in order to optimally engage with an activity. Clear feedback helps the musician to adapt his performance to the context, which is itself largely dependent on his experience, skills and knowledge. The person has an immediate feedback of how well his or her action is progressing (Nakamura & Csikszentmihalyi, 2002); at any time, he or she can evaluate whether the previous sequence was done well or not. Our pianist will probably have a rather good track of whether her playing was good or not. A false note, disharmony, uncontrolled change of rhythm or inappropriate colour of tone will be immediately heard and recognized as a failure. Further, a perfectly performed piece will be instantly perceived as well. According to these contextual cues, the pianist will be able to adjust her action, to correct, highlight certain moments or to bedim them. Immediate feedback is also closely related to the notion of challenge-skill balance. New feedback (either external or subjective) will provide new environmental cues on the relationship between the person’s actual skills and contextual challenges. The continuity of immediate feedback is dependent on attentional involvement as well. Without paying close attention to what we are doing, we cannot really have an idea of how well we are doing. In this sense, we can imagine that this instant feedback mediates between skill-challenge balance on the one side, and attentional involvement on the other side.

### Core Processes

Core processes are the mediating and/or moderating mechanisms that transform inputs into outputs. In our model, those processes designate instantiations of certain fundamental cognitive mechanisms. If we imagine that *inputs* are the fuel for flow, then we could comparably say that *core processes* are ignition to the flow engine. Our schema of flow mechanics includes two core processes: (1) attention, and (2) motivation.

#### Attentional Process

The first core process in our model is attentional involvement. The flow experience relies on a unique configuration of attentional mechanisms. The attentional involvement was found to be a mediating variable for the relationship between optimal challenge and enjoyment, and the relationship between competence valuation and enjoyment. Using Experience Sampling Method, Abuhamdeh and Csikszentmihalyi (2012a) have examined the relationship between challenge and enjoyment on undergraduate students. The measure comprised questions concerning enjoyment (e.g., were you enjoying yourself?), balance of challenges and skills (e.g., how challenging was the activity?), competence valuation (e.g., was doing well important to you?). Their analysis indicated that attentional involvement accounts for 62% of the total effect between skill-challenge balance and enjoyment. Further, the attentional involvement fully mediated the relationship between competence valuation and enjoyment, accounting for 80% of the total effect. This means that when attentional involvement increases, a big part of attentional resources are devoted towards the task, and features of activity engagement therefore can be experienced more fully (Abuhamdeh & Csikszentmihalyi, 2012a). This finding highlights the importance of attentional involvement in intrinsic motivation processes.

In this paper, we have allowed ourselves to go a step further in discussing the nature of this attentional involvement. The component of attentional involvement in flow is unlikely to correspond to *sustained* or *directed attention* (e.g., Posner, 1994) – those that enable maintenance of vigilance, selective and focused attention response persistance, and effort despite changing conditions. Otherwise, it would not be described as a phenomenon of effortless attention (see Bruya, 2010). Hence, the attentional involvment in flow is closer to some less costly, more implicit attentional mechanisms with eventual ad-hoc interventions of certain control mechanisms.

In our flow model, the attentional component is composed of two sub-components: automatic attention – referring to implicit investment in the task, and executive attention – referring to explicit intervention of executive control. Dietrich (2004), for instance, proposes a neurocognitive account of flow as a special case of transient hypofrontality – a state where the focused part of the brain (explicit system), which is responsible for top-down processes, gets a rest while other parts and functions, reponsible for bottom-up processes (implicit system), become more predominant. Dietrich (2004) differentiates two distinct information processing systems: (1) the explicit system and, (2) the implicit system. Dietrich (2004, p. 746) proposes a thesis that classifies the flow state as a period where a

highly practiced skill that is represented in the implicit system’s knowledge base is implemented without interference from the explicit system. It is proposed that a necessary prerequisite to the experience of flow is a state of transient hypofrontality that enables the temporary suppression of the analytical and meta-conscious capacities of the explicit system.

Flow would then be defined as a *„state of hypofrontality with the notable exception of executive attention, which enables the one-pointedness of mind by selectively disengaging other higher cognitive abilities of the prefrontal cortex“* (Dietrich, 2004, p. 757).

**Figure 2:**
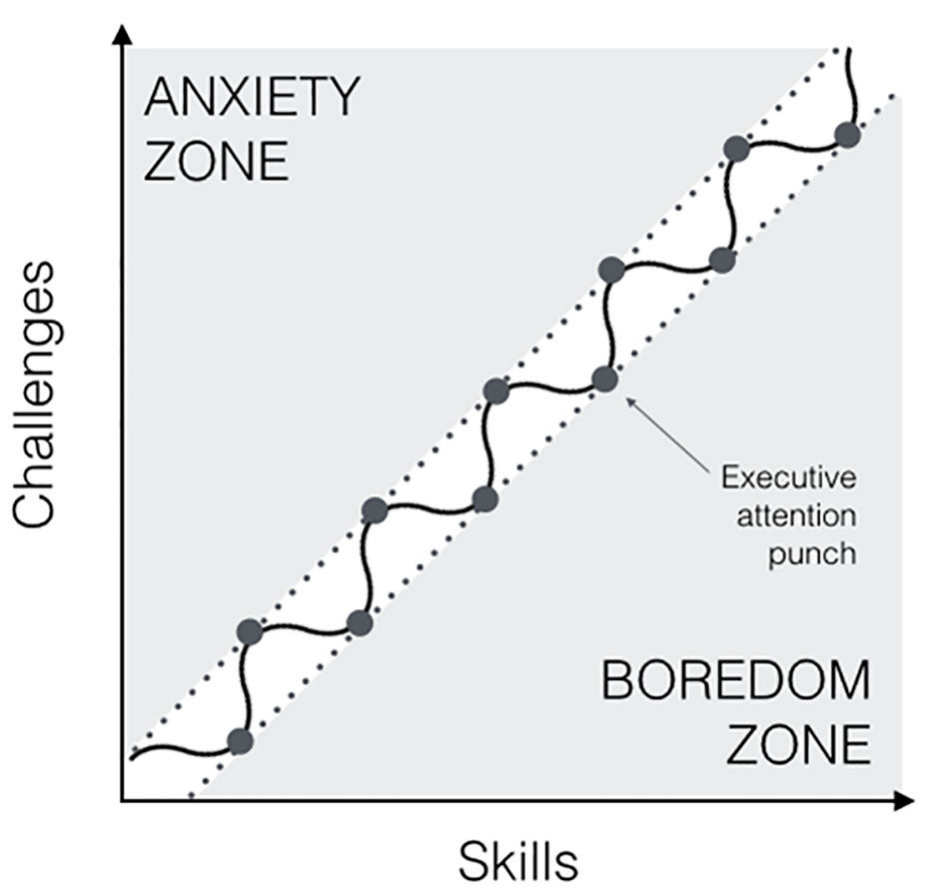
The flow channel and micro-disbalance between skills and challenges.

Schematically, if we imagine flow as a constant micro-disbalance between perceived skills and challenges, we could represent it as an upward, wavy motion through the flow channel (see Figure 2).

Inside the channel, the person would function on an autopilot, in a state of hypofrontality. However, once the skill has evolved, the trend will head downwards to *boredom* zone - which potentially brings task-irrelevant thoughts (Smallwood et al., 2004). In order to maintain the flow, an executive punch is needed such that fresh challenges readjust, matching these newly strengthened skills. Conversely, if the challenge exceeds the skills, drawing the person into the anxiety zone, a special effort is needed to bring the requirements back into the channel where they match the skills. Overall, the attentional involvement in the flow process mostly corresponds to automatic processing where the person feels she operates without explicit effort. This suggests that the prefrontal cortex is not required for the successful execution of the task (Dietrich, 2004), in the short term. In the long term, this state of hypofrontality is occasionally interrupted by an executive intervention that aims restoring the implicit, hypofrontal state.

Recent, but scarce literature about neural correlates of flow yield unclear and contradicting neuroimaging results when it comes to hypofrontality hypothesis. On the one hand Ulrich, Keller, Hoenig, Waller, and Grön (2014) find decreased activity in medial prefrontal cortex, implying that there is decreased self-referential processing while in flow. On the other hand, Harmat et al. (2015) find no association between cortical oxygenation and flow, and therefore no support that flow is related to a state of hypofrontality. However, it is very important to point out methodological and instrumental differences between these two studies: in terms of the administered task (mental arithmetic task in the first case and a tetris game in the second), neuroimaging instrument (magnetic resonance imaging versus functional near-infrared spectroscopy) and experimental subjects (exclusively male sample versus exclusively female sample). The great methodological discrepancy between the studies makes it very risky to conclude on the neural basis of hypofrontality in either way. More future studies are required in this field in order to gain better understanding of neural basis of flow process.

#### Motivation

To be motivated means to be moved to act, to accomplish or do something. “A person who feels no impetus or inspiration to act is thus characterized as unmotivated, whereas someone who is energized or activated toward an end is considered motivated” (Ryan & Deci, 2000, p. 54). Being involved in an activity providing flow requires a certain kind and certain level of motivation that moves the doer’s will to continue being invested in the activity. Initial clear proximal goals allow the emergent higher-order motivation to take place and to ignite the flow mechanics. Once in place, motivation (together with attention) allows one to maintain the momentum in flow activities.

There are essentially two types of motivation: (1) intrinsic motivation, which refers to being involved in an activity because it is interesting itself or enjoyable, and (2) extrinsic motivation, which refers to doing something because it leads to a detachable outcome (Ryan & Deci, 2000).

Intrinsic motivation means being motivated for an activity purely for the sake of that activity itself (Deci & Ryan, 1985; Lepper, Greene, & Nisbett, 1973). People pursue intrinsically motivated activities voluntarily, when external constraints are absent (Deci & Ryan, 1985; Harackiewicz, Manderlink, & Sansone, 1984). These activities are pursued for the enjoyment of experience (Abuhamdeh & Csikszentmihalyi, 2012a). Amabile (1996) defines as intrinsic any motivation coming from the person’s positive reaction to qualities of the task itself, while defining extrinsic motivation as any motivation that arises from sources outside of the task. According to this author’s Intrinsic Motivation Hypothesis, the intrinsically motivated state is conductive to creativity, whereas the extrinsically motivated state is mostly detrimental to creativity with very few exceptions concerning external motivators, in service of intrinsics, that are perceived as informational, enabling or socially empowering (e.g., recognition). Deci (1971) also found that extrinsic motivators do not all work the same way and not all of them hinder intrinsic motivation: for example, rewards such as social approval do not seem to affect a person’s intrinsic motivation as negatively as monetary rewards do (Deci, 1971).

Inasmuch as flow activity is autotelic (done for the sake of doing) and associated with creative achievements, it is considered to involve intrinsic motivation. Motivation, together with activity type, was found to be a moderating factor in a relation of perceived challenge and reported enjoyment (Abuhamdeh & Csikszentmihalyi, 2012b). Moreover, the link between challenge and enjoyment was bigger for intrinsically motivated, goal-directed activities than for non-intrinsically motivated, goal-directed activities and intrinsically motivated, non-goal directed activities.

The involvement of intrinsic motivation in flow is also consistent with the absorbing aspect of the flow experience: although flow activities can be motivated by a spark of some kind of extrinsic goal in the contextual precondition factors, during the task execution (or core process) there is no space in consciousness for contemplation of extrinsic goals. In line with this argument, Amabile (1996) draws a conceptual link between the type of motivation and attentional involvement in order to give an account for the underlying cognitive mechanism. The difference between extrinsic and intrinsic motivation is compared to the difference between divided and undivided attention to task-relevant information and to the task itself. Attentional ressources are not limitless. An extrinsically motivated person will use at least some of those resources in monitoring if the action is meeting the extrinsic goal or not (Amabile, 1996). Consequently, extrinsically motivated people will be less able than intrinsically motivated people to completely focus their attention to the task and task-relevant environmental cues. Narrowly linked to the attentional process, intrinsic motivation can be seen as a catalyst of the flow process. Therefore, in our model, intrinsic motivation appears as a very important moderating variable of the attentional mediation between inputs and outputs.

### Outputs

Psychological outputs from reaching the optimal experience follow three sets of outcomes: (1) Subjective experience of absorption, related to phenomenon of hyper focus, lack of reflective self-awareness and time distortion, (2) positive affects such as satisfaction, pleasure, joy, feeling alive; and (3) results, the fruits of invested effort such as relative performance, creativity and other forms of achievements. Outcomes of flow may nourish the inputs in the sense of creating a virtuous circle of flow.

#### Absorption

While attentional involvement refers to a core process in flow, composed of two mechanisms (automaticity and executive attention), the absorption refers to a subjective feeling resulting from the flow process. Experience of absorption covers the following characteristics: lack of self-awareness, hyper focus and distortion of temporal experience. We argue that those three characteristics are close enough to be grouped into one output and for the sake of parsimony, we decided to group them under the umbrella term *absorption.*

Tellegen and Atkinson (1974) interpret absorption as a disposition for having episodes of “total” attention that fully engage one’s representational ressources. They suggest that the type of attention involved in absorption experience is centered, amplifies the experience of one part of reality, involves a full commitment of available perceptual, motoric, imaginative and ideational ressources to a unified representation of the attentional object (Tellegen & Atkinson, 1974). In our view, this dispositional account of absorption seems closely related to Dietrich’s (2004) hypothesis of hypofrontality, on which we rely to elaborate on the attentional processes in action in the core process of flow.

Due to being focused, the person experiencing flow has neither time nor cognitive resources to invest in auto-reflexion. The activity becomes spontaneous; the self becomes absent from the consciousness. This means that while flowing, an individual temporarily pauses the thoughts that refer to self – how do I look, am I hungry, does my body hurt, etc. In flow, “one acts with a deep but effortless involvement that removes from awareness the worries and frustrations of everyday life” (Csikszentmihalyi, 2008, p. 49). This is true for most of the domains except maybe for some autoreflexive activities such as certain forms of meditation or prayer. Due to the lack of research on flow in this kind of activities, where the reflexion upon the self is in the heart of the task itself, we cannot make further assumptions.

Narrow, task-related hyperfocus characterizes the flowing experience. The person gets so intensely immersed in the activity that awareness and action merge in the present moment: here and now. During performance, the pianist is so immersed in playing that not much can get her out her element: a cell phone ringing in the audience, the sound of rain outside, the memory of her grandmother who passed away two days ago, etc. The contrary of hyper focus is *psychic entropy*, a disorganization of the self that impairs its effectiveness. The absorption corresponds to hypofrontality (Dietrich, 2004) where the explicit system is unburdened or inhibited.

Early research into the psychological aspects of time demonstrated that human temporal perception was not a simple chronometric record of reality (Hancock & Weaver, 2005). While flowing, a person is deeply involved attention-wise. Consequently, the perception of time passing can be significantly altered. When flowing, people usually report that time seems to pass very quickly (Nakamura & Csikszentmihalyi, 2002). However, this might not be completely generalizable to all domains of activities. The flow in strictly time-dependent activities such as competitive running might be an exception because the awareness of time passing constitues the structure of the task itself.

In conclusion, we gather in this first output of flow process the subjective experience of absorption, the lack of self-awareness, hyper focus and distortion of temporal experience. This series of phenomena are directly related to the attentional mechanism of hypofrontality highlighted in flow core processes.

#### Positive Affects

Research investigating the nature of autotelic experiences by consulting rock climbers, chess players, dancers and other professions showed that the enjoyment was the main reason why individuals pursued the activity (Csikszentmihalyi, 1975/2000, as cited in Nakamura & Csikszentmihalyi, 2002). The genuine enjoyment that surgeons, rock climbers, and other professionals routinely find in their activities depict how an organized set of challenges and a corresponding set of skills result in optimal experience (Nakamura & Csikszentmihalyi, 2002).

Research using Experience Sampling Method in order to test flow has confirmed that subjects report the best subjective experiences when both perceived challenges and skills are high and well balanced. When flowing, they report feeling more active, alert, concentrated, higher levels of happiness, satisfaction, and creativity—although not necessarily more cheerful or sociable (Carli, 1986; Massimini, Csikszentmihalyi, & Carli, 1987; Nakamura, 1988; Wells, 1988).

Seligman and Csikszentmihalyi (2014, p. 8) make a clear distinction between

positive experiences that are pleasurable and those that are enjoyable. Pleasure is the good feeling that comes from satisfying homeostatic needs such as hunger, sex, and bodily comfort. Enjoyment, on the other hand, refers to the good feelings people experience when they break through the limits of homeostasis – when they do something that stretches them beyond what they were – in an athletic event, an artistic performance, a good deed, a stimulating conversation.

#### Task Achievements

Task achievements include feeling of control and performance (e.g., productivity and creativity). Merged into one output, they represent objective (productivity) and subjective (feeling of control) performance in a given task.

Adaptive goal-directed behaviour includes monitoring of ongoing actions and performance outcomes, and resulting adjustments of learning and behaviour (Ridderinkhof, Ullsperger, Crone, & Nieuwenhuis, 2004). Thanks to the balance between perceived skills and perceived challenges and attentional involvement, the person experiencing flow has an impression of being in control of the situation. The sense of control is one of the main indices of flow (Csikszentmihalyi, 2000). The idea that the control of consciousness improves the quality of life has been present for a long time: in almost every Eastern spiritual tradition (Csikszentmihalyi, 2008, p. 20).

This control of the consciousness sometimes reminds one very much of *mindfulness meditation*, “the awareness that emerges through paying attention, on purpose, and nonjudgmentally to the unfolding of experience moment by moment” (Kabat-Zinn, 2003, p. 145, as cited in Luken & Sammons, 2016). Research has shown a significant relationship between flow experiences and mindfulness (e.g., Wright, Sadlo, & Stew, 2006). Kee and Wang (2008) found that higher levels of mindfulness in university athletes related to higher levels of flow components, such as: the balance between skills and challenges, merging of action and awareness, concentration, clear proximal goals and loss of self-consciousness (Kaufman, Glass, & Arnkoff, 2009). In an interview with an art and design student, Allen and Loughnane (2016, p. 684) offer a vivid illustration of mindfull involvement:

Speaking from an artist's point of view, you can get so involved in being present with the creative process that involvement with an art activity can be hugely stress relieving; you are so focused on the present moment that nothing else permeates the process. It's an effective tool for mindfulness, I personally find.

However, the experience of flow considerably diverges from mindfulness. According to Dane’s (2011, pp. 1001-1002) classification in terms of attentional scope (large versus narrow) and the focus on the present moment (high versus low), there are four types of of attentional states: (1) mindfulness (large attentional scope and high focus on the present moment), (2) absorption/flow (narrow attentional scope and high focus on the present moment), (3) distraction/mind-wandering (large attentional scope and low focus on the present moment), and (4) prospective thinking/counterfactual thinking (narrow attentional scope and low focus on the present moment). In line with this categorization, flow and mindfulness both correspond to high levels of focus in the present moment, but they contrast in terms of attentional scope. While mindfulness refers to a maximum openness to all stimuli (internal and external), flow covers a very narrow focus field, therefore often leading to the lack of self-consciousness. This relative lack of reflective self-consciousness makes these two phenomena incompatible in a given moment (Sheldon, Prentice, & Halusic, 2015). Therefore, mindfulness cannot be an output of the flow process.

Some literature suggests there is a positive relationship between flow and performance, especially in learning settings (e.g., Engeser, Rheinberg, Vollmeyer, & Bischoff, 2005; Schüler, 2007; Schiefele & Rheinberg, 1997, as cited in Schüler & Brunner, 2009), artistic and scientific creativity (e.g., Perry, 1999; Sawyer, 1992, as cited in Schüler & Brunner, 2009). Engeser and Rheinberg (2008) found that flow predicted academic performance in two out of their three studies (learning for an obligatory course in statistics and learning in a voluntary French class). According to Engeser and Rheinberg (2008), there are at least two good reasons why flow should be related to performance. First, flow is a phenomenon of high functioning that should in itself encourage a good performance. Furthermore, individuals experiencing flow feel more motivation “to carry out further activities, and in order to experience flow again, they will set themselves more challenging tasks” (Bakker, Oerlemans, Demerouti, Slot, & Ali, 2011). Likewise, Schüler and Brunner’s (2009) observations suggested that flow experience during a marathon is associated with the motivation for future running but not with the present race performance. “Flow functions as a reward of the running activity, which leads to the desire to perform the activity again” (Schüler & Brunner, 2009, pp. 173). This body of results is in line with the argument that the links between flow and performance may be both direct (with performance resulting from the flow process) and indirect (with feedback loops fueling either the skill-challenge balance or the intrinsic motivation core process). However, we may also mention that these potential interrelations between flow and performance are not always supported empirically: divergent and inconsistent results were reported in the domains of sport (Bakker et al., 2011; Jackson, Thomas, Marsh, & Smethurst, 2001), music (Iusca, 2015), and work setting (Demerouti, 2006). These inconsistencies might be due to various reasons: the big disparity of nature of tasks measured, heterogeneity of flow assessment methods and plurality of performance measurements.

Finally, there is some empirical evidence that flow is related positively to creativity. MacDonald, Byrne, and Carlton (2006) used the Experience Sampling Method to measure flow in tasks of musical group composition. Their results clearly show higher levels of flow are associated to higher levels of creativity (MacDonald et al., 2006). Similar findings appear in the domain of work psychology. Namely, Zubair and Kamal (2015) gathered data from 532 workers in software companies discovering that work related flow was a strong predictor of employee creativity (Zubair & Kamal, 2015). On the other hand, the research in visual arts is somewhat less clear. Flowing participants performing creative mental synthesis to simulate creative process of drawing exhibited an affect improvement in visual creativity (Cseh, Phillips, & Pearson, 2015). In their experiment (Cseh et al., 2015) using creative mental synthesis task (Finke & Slayton, 1988), researchers found that the change in affect was related to productivity and self-rated creativity. However, it was not linked to other objective or subjective performance measures evaluated by judges. Even though flow, measured by pre-task and post-task questionnaires was not related to all performance measures, it was notably correlated with self-related creativity. This study aiming to understand flow in visual creativity concludes that flow motivates perseverance towards eventual excellence rather than providing straight cognitive improvement (Cseh et al., 2015).

## Conclusion

Flow states have escaped, in many ways, the attention of cognitive psychology and neurosciences. Mostly studied in the context of correlational studies, with quite limited data collected in the context of controlled experiments, flow seems to be implicitly considered as an applied concept from positive psychology or as an esoteric discipline. Our main aim in this paper was to try to integrate it into the framework of mainstream cognitive psychology and relate it to the major cognitive functions of human psyche.

The flow engine framework explains the relationship between flow characteristics using an engine metaphor. Skill challenge balance, clear proximal goals and immediate feedback fuel the process and represent necessary logical requirements for flow. Skill-challenge balance allows attention to be used in an optimal way. Immediate feedback and clear proximal goals fuel the attention, which in turn gives an update about new skills and challenges relation. These combustibles are then ignited by strokes in the cylinders – the core processes. Like interdependent sparks, attentional involvement, composed of automaticity and executive attention, and intrinsic motivation start the dynamism of this flow machine. Adequate attentional involvement results in outcomes linked to absorption. The overall process corresponds to a moderated mediation between inputs and outputs, with attention (automaticity and executive attention) as mediator and intrinsic motivation as moderator. As a result of a well-done task, task achievements occur often (but not always) as an outcome of flow process. When this happens, task-achievement results immediately in an update of skill-challenge balance, modifies proximal goals, multiplies positive affect and therefore reinforces the motivation for future engagement in the task. Unlike Landhäusser and Keller (2012), we focus on putative dynamic and causal relations between flow components involving generic attentional and motivational processes.

One of the important implications of this model is that flow is regarded as a processing mechanism rather than a mere mental state or performance state (e.g. Jackson et al., 2001). This implies that existing indicators of flow might not be optimally adapted in regard to the nature of phenomena. This means that actual flow-scales and tools capture flow components retrospectively or quasi-retrospectively, as if they were of the same essence. Our model does involve these flow dimensions and sorts logically their structural order in a dynamic and interdependent framework. It holds that flow represents a macro-process embracing two core cognitive processes: (1) attention (automaticity with sparks of executive attention) and (2) intrinsic motivation. From this perspective, it appears necessary to step back and review how these two processes function in the context of optimal experience and how their variation modulates the episodes of flow.

## Future Directions

Firstly, the model proposed here should be empirically validated. Actual lack of methods to test for causal effects of flow experiences should be overcome by novel approaches of examining flow.

Despite 30 years of empirical attention that has been given to the flow research, nearly all of it has been correlational in nature (Csikszentmihalyi, 1975; Moller, Meier, & Wall, 2010). The concept itself originated from observational and interview-based research on creativity (Moller et al., 2010). This heavy reliance on correlational methods results is a major weakness, resulting in unresolved questions about causality and the direction of relations observed between flow, contextual factor and personality traits (Spencer, Zanna, and Fong, 2005; as cited in Moller et al., 2010). In order to validate our model and causal relation between inputs, core processes and outputs, experimental research is needed. By experimentally varying the inputs, the causal relationship with outputs, as well as the hypothesized effects of mediators and moderators, can be assessed. The inputs could be manipulated through, for example varying skills (via training), challenges (rapidity or difficulty of a task), and feedback (the transparency of the task progression structure). Video games seem to be a very convenient kind of task for experimentally testing such hypotheses (e.g., Cabo, Kleiman, McCauley, & Parks, 2004; Moller, Csikszentmihalyi, Nakamura, & Deci, 2007; Parks & Victor, 2006).

To validate the moderated mediation, it is required to accurately measure both core processes. Situational intrinsic motivation can be easily assessed through standardized scales such as SIMS (16-item Situational Motivation Scale by Guay, Vallerand, & Blanchard, 2000). On the other hand, the fine interplay of the two attentional elements: automaticity and executive attention, seems more challenging to grasp, but not impossible. As suggested in the hypofrontality framework, automaticity might be measured through neurophysiological indicators of the decrease in the need for effortful control (e.g., Poldrack et al., 2005; Kübler, Dixon, & Garavan, 2006). Executive attention, which helps in coping with irrelevant or distracting items while experiencing flow, might be assessed thanks to the presence of such interfering stimuli. For instance, if the flow-generating activity is conducted while visual or auditory irrelevant stimuli are delivered, the effectiveness of coping with distraction may be measurable through the habituation of electrodermal orienting response (Naveteur, Buisine, & Gruzelier, 2005; Waters, McDonald, & Koresko, 1977), antisaccade paradigm or implicit learning of unattended stimuli (Engle, 2002).

We can easily think of a tangible example of an experimental scenario taking into account most of the framework’s components using Candy Crush game. Practically speaking, the participants can be given a match-three puzzle video game with occasional interference of banner ads that appear on the screen during the gameplay. In that way, the players need to use their executive attention in order to ignore the ads. Varying skill-challenge relation could be done by designing boredom, adaptive (flow), and overload conditions - like in Keller and Bless (2008). While playing the Candy Crush game with one hand on a smartphone or tablet, the other free hand can be used to measure electrodermal response to distracting ads. At the end, performance achievement (e.g. number of puzzles completed, points gained) can be collected, standardized scales assessing intrinsic motivation, absorption and positive affect can be administrated, as well as a recognition test of the ads (supposedly low in flow conditions) in order to evaluate the effectiveness of executive attention. This is just one idea among numerous empirical possibilities that can be envisioned for the purpose of examining and validating the conceptual framework.

Finally, since its formalisation, there has been rich and vast research concerning flow in individual settings. Nevertheless, the majority of human activity is social and happens in a group setting. There has been extremely little research about flow in group-like, team-based, collective or interdependent activities (e.g., Salanova, Bakker, & Llorens, 2006; Sawyer, 2003, 2012; Walker, 2010). Therefore, it would be highly valuable to explore the phenomenon of flow in groups. Effort has been made to study flow in certain group tasks (e.g., school activities and team sports), but mostly treating the individual as the focus of analysis (Nakamura & Csikszentmihalyi, 2002). Thus, the question arises: is there something similar to flow in groups and how does it work? In the studies of Csikszentmihalyi (2008) on the quality of daily experience “it has been demonstrated again and again that people report the most positive moods overall when they are with friends”. “A key characteristic that the flow model shares with other contemporary theories is *interactionism* (Nakamura & Csikszentmihalyi, 2002). Rather than focusing on the person, abstracted from the context (i.e., traits, personality types, stable dispositions), flow research has emphasized the dynamic system composed of person and environment, as well as the phenomenology of person-environment interactions” (Nakamura & Csikszentmihalyi, 2002). In the case of group flow, social psychology theories might be explored in order to understand group processes leading to optimal collaboration.
